# CRISPR-based strategies for targeted transgene knock-in and gene correction

**DOI:** 10.12703/r/9-20

**Published:** 2020-12-04

**Authors:** Cia-Hin Lau, Chung Tin, Yousin Suh

**Affiliations:** 1 Department of Biomedical Engineering, Academic 1, 83 Tat Chee Avenue, City University of Hong Kong, Hong Kong; 2 Department of Obstetrics and Gynecology, Columbia University Irving Medical Center, 630 West 168th Street, New York, NY 10032, USA; 3 Department of Genetics and Development, Columbia University Irving Medical Center, 630 West 168th Street, New York, NY 10032, USA

**Keywords:** Gene correction, gene knock-in, gene replacement, genetic variant, single-nucleotide polymorphism, targeted integration

## Abstract

The last few years have seen tremendous advances in CRISPR-mediated genome editing. Great efforts have been made to improve the efficiency, specificity, editing window, and targeting scope of CRISPR/Cas9-mediated transgene knock-in and gene correction. In this article, we comprehensively review recent progress in CRISPR-based strategies for targeted transgene knock-in and gene correction in both homology-dependent and homology-independent approaches. We cover homology-directed repair (HDR), synthesis-dependent strand annealing (SDSA), microhomology-mediated end joining (MMEJ), and homology-mediated end joining (HMEJ) pathways for a homology-dependent strategy and alternative DNA repair pathways such as non-homologous end joining (NHEJ), base excision repair (BER), and mismatch repair (MMR) for a homology-independent strategy. We also discuss base editing and prime editing that enable direct conversion of nucleotides in genomic DNA without damaging the DNA or requiring donor DNA. Notably, we illustrate the key mechanisms and design principles for each strategy, providing design guidelines for multiplex, flexible, scarless gene insertion and replacement at high efficiency and specificity. In addition, we highlight next-generation base editors that provide higher editing efficiency, fewer undesired by-products, and broader targeting scope.

## Introduction

Clustered regularly interspaced short palindromic repeats (CRISPR) has revolutionized the field of transgenic research and human gene therapy^1–6^. Using CRISPR, a specific genetic variant associated with human disease can be introduced into the genome, or a mutation in the genome can be replaced with a wild-type allele, at will by design in cells and organisms, respectively, for gene knock-in or gene correction^7–12^. The CRISPR-modified cellular and animal models then can be studied to uncover mechanisms underlying human disease and for drug discovery^13–15^. Therefore, CRISPR holds tremendous translational potential. CRISPR technology has been explored for *in vivo* gene therapy to treat a wide range of human hereditary diseases^16–18^ as well as *ex vivo* gene therapy to treat blood disorders, cancers, and immune-related diseases by genetically modifying the patient’s cells outside their body^19–23^. The last few years have seen enormous advances to improve the performance and maximize the potential of CRISPR-mediated gene insertion and replacement for disease modeling and gene therapeutics.

The strategies for transgene knock-in and gene correction are generally classified into homology-dependent and homology-independent. Homology-directed repair approaches require the presence of both CRISPR/Cas9 and a DNA donor repair template. The repair template can be in the form of circular double-stranded plasmid DNA^24–27^, single-stranded donor oligonucleotide (ssODN)^28–30^, linear double-stranded polymerase chain reaction (PCR) fragments^31,32^, or the homologous sequences of the intact sister chromatid^33^. Depending on the forms of repair template and CRISPR system used, homology-mediated gene insertion and replacement are carried out via specific DNA repair pathways such as homology-directed repair (HDR)^24,26,33^, synthesis-dependent strand annealing (SDSA)^28,34^, microhomology-mediated end joining (MMEJ)^35,36^, and homology-mediated end joining (HMEJ)^27,37–39^ pathways. In homology-independent approaches, transgene knock-in and gene correction are achieved with CRISPR/Cas9 in the absence of DNA donor repair templates. In this case, the error-prone non-homology end joining (NHEJ) repair pathway is commonly used to repair CRISPR-mediated double-stranded breaks and to incorporate a mutagenic sequence into the genome^40,41^.

As alternatives, base editing^42–45^ and primer editing^46^ are used to perform gene correction and replacement by directly converting nucleotides in genomic DNA without damaging the DNA or requiring donor DNA. Various base-editor variants have also recently been engineered to provide higher editing efficiency, fewer undesired by-products, and broader targeting scope. Simultaneous substitution of multiple nucleotides has been achieved by fusing Cas9 nickase (Cas9n) to a DNA polymerase^47^ or a reverse transcriptase (RT)^46^. To avoid permanent deleterious effects caused by unanticipated mutagenesis and complex chromosomal rearrangements to genomic DNA, CRISPR is employed to edit the endogenous RNA transcripts containing pathogenic mutations^48–50^.

Given the explosive popularity and rapid evolution of CRISPR technology in transgenic research and human gene therapy, the purpose of this review is to provide a comprehensive summary on various, recently developed CRISPR-based strategies for controlled and desired transgene knock-in and gene correction as a quick reference for those who are new to this fast-moving field. We illustrate and highlight the mechanisms of action and key concepts for each transgene knock-in and gene correction strategy. We elaborate design guidelines for scarless gene insertion and replacement at high efficiency and specificity. We also critically discuss the applications and pros and cons of each strategy. Finally, we briefly discuss how engineered CRISPR variants and alternate donor template designs have improved the efficiency, specificity, editing window, and targeting scope of transgene knock-in and gene correction.

## Homology-dependent gene knock-in and gene correction strategies

### Homology directed repair–based approaches

**Figure 1.**
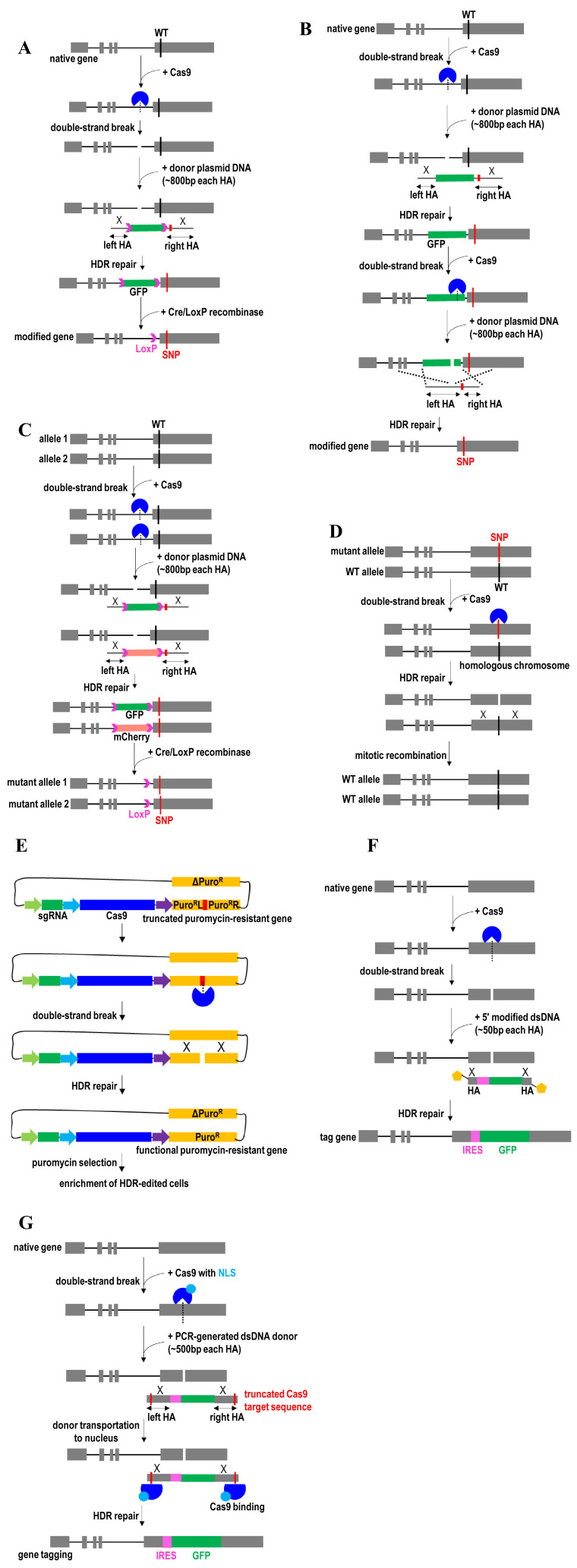
Homology directed repair (HDR)-mediated gene knock-in and gene correction strategies. (**A**) Exonic SNP knock-in by CRISPR/Cas9-mediated integration of a selection marker in intron and an SNP in exon, followed by Cre/LoxP removal of the selection marker. (**B**) Exonic SNP knock-in by CRISPR/Cas9-mediated integration of a selection marker at intron and an SNP at exon, followed by CRISPR/Cas9-mediated removal of the selection marker. (**C**) Biallelic SNP knock-in by CRISPR/Cas9-mediated integration of two different fluorescent reporter genes, followed by Cre/LoxP removal of these two reporter genes. (**D**) Gene correction via CRISPR/Cas9-mediated mitotic recombination. (**E**) Enrichment of HDR-edited cells by a universal surrogate reporter plasmid. (**F**) Exonic SNP knock-in using Cas9 ribonucleoprotein complexes and 5′-modified linear dsDNA donors. (**G**) Transgene knock-in facilitated by adding truncated Cas9 target sequences to the 5′ end of left homology arm and 3′ end of right homology arm in plasmid donor. GFP, green fluorescent protein; HA, homology arm; HDR, homology-directed repair; IRES, internal ribosome entry site; NLS, nuclear localization sequence; Puro, puromycin; SNP, single-nucleotide polymorphism; WT, wild-type.

HDR mediated by homologous recombination is one of the most commonly used methods to introduce a genetic mutation into the genome (gene knock-in). It allows desired and controlled genetic modifications in the genome. This HDR approach requires the presence of a DNA repair template, usually in the form of circular plasmid DNA. However, HDR activity can be improved using the linearized plasmid with the short 5′ backbone overhang^51^. To introduce an exonic mutation, CRISPR/Cas9 ribonucleoprotein (RNP) complex is co-delivered with the plasmid DNA donor containing two homology arms (~800 bp each arm) flanking the mutated sequence into the cell (Figure 1A). As an option, a fluorescent tag can be added to the plasmid DNA donor to facilitate the selection of edited cells. To ensure efficient mutational knock-in, one should try to locate the mutated sequence as close as possible to the 3′ end of the left homologous arm or the 5′ end of the right homologous arm. CRISPR/Cas9 first induces a double-strand DNA break at the target intronic region, usually cleaving at 3 or 4 nucleotides upstream of the protospacer adjacent motif (PAM) sequence. The left and right homology arms should flank the CRISPR/Cas9 cleavage site. HDR-based cellular DNA repair machinery then is activated to repair the CRISPR/Cas9 cleavage site. The desired genetic variant and the fluorescent marker in the donor repair template are introduced into the genome via homologous recombination. The single-cell clones of these edited cells are flow-sorted on the basis of their fluorescent marker expressions. Finally, this fluorescent marker gene at the intronic region is removed by the overexpression of Cre/LoxP recombinase prior to functional analysis. However, a copy of a short LoxP element would remain within the intron. This knock-in approach is useful for characterizing protein-coding variants (for example, non-synonymous single-nucleotide polymorphism [SNP] or missense mutation)^24,52,53^. Although an intronic variant can also be introduced to the genome via this approach when the exon sequence is short, a scarless gene-editing outcome is preferred.

For introducing non-coding variants within the regulatory elements (for example, a mutation in the promoter, enhancer, or untranslated region), a clean mutational knock-in or scarless genome-editing approach is recommended. For example, the fluorescent marker gene at the intronic region is removed without the addition of any exogenous sequence by designing the single guide RNA (sgRNA) to target the fluorescent marker gene (Figure 1B). Upon cleavage of the CRISPR/Cas9 second target site, the edited allele uses the second plasmid donor bearing only an SNP (without fluorescent marker transgene) to mediate homologous recombination^26^. After the two-step editing process, the edited cells with no fluorescent protein expression are flow-sorted and single-cell-expanded for characterization. Alternatively, marker-free genome editing can be achieved by using a highly efficient HDR approach that was developed recently^28,54^. This approach uses electroporation to deliver adeno-associated virus (AAV) 6-mediated DNA repair template and Cas9 protein complexed with chemically modified sgRNA. As an alternative, the repair template can be delivered as an ssDNA donor. As the majority of cells are successfully edited, no selection marker (for example, fluorescent marker or antibiotic selection) is required to screen a large number of clones to identify biallelic edited clones. Therefore, this scarless genome-editing approach is particularly useful for therapeutic applications in hard-to-transfect cells such as human pluripotent stem cells^54^, adult stem cells^28,55^, and primary immune cells^56^.

Because some diseases are caused by the homozygous mutations^57–60^, efficient site-specific gene correction of both alleles is needed. Moreover, a single-allele perturbation might not be sufficient to cause significant functional alteration of a given gene, especially for disease-associated genetic variants such as non-synonymous SNPs. Thus, transgenic cells with biallelic gene manipulation are usually preferred over monoallelic, even for SNPs in a heterozygous form, to be able to detect the functional impact of the variants on the activity and molecular phenotypes of the targeted gene product. However, owing to the inefficient HDR events, it is often technically more challenging to screen transgenic cells with targeted mutational knock-in at both alleles than monoallelic gene editing. Therefore, a CRISPR/Cas9-mediated biallelic genome targeting with a dual surrogate reporter-integrated donor system was recently developed to facilitate the selection and enrichment of biallelic-modified cells^61^. This approach uses a CRISPR/Cas9-mediated homologous recombination system and two different sets of reporter transgene (Figure 1C). For example, CRISPR/Cas9 is first used to induce DNA double-strand breaks near the desired exonic mutation at both alleles. Then each reporter transgene (green fluorescent protein [GFP] or mCherry) is integrated into each allele. The enhanced GFP (eGFP) and mCherry double-positive cells then are sorted and expanded. The Cre/LoxP recombination system is used to remove these two fluorescent selection marker transgenes in these biallelic-modified cells prior to functional analysis. The use of this strategy can avoid time-consuming and tedious screening procedures to identify biallelic-modified cells, thereby enabling high-throughput characterization of non-synonymous mutations on gene function. A caveat of this biallelic modification strategy is that it cannot be used for essential genes that can affect cell viability. Extensive subcloning and prolonged passage of reporter-inserted clones may also lead to subtle phenotype deviations from the original parental cell line.

Gene correction has also been achieved via mitotic recombination using the endogenous wild-type allele on the homologous chromosome as a template (Figure 1D)^33^. In this case, the sgRNA is designed to target the mutant allele. CRISPR/Cas9-induced DNA breaks in the mutant allele then are repaired through homologous recombination using the wild-type allele on the homologous chromosome as a template. This strategy has been employed for gene drive applications in a polyploid organism^62–65^. In the case of gene drive, CRISPR/Cas9 possibly along with a payload transgene (desired trait) is first inserted into one of the wild-type alleles in the genome. The insertion is done using CRISPR/Cas9-mediated homologous recombination. Expressions of CRISPR/Cas9 then trigger another round of homologous recombination in the cell to modify the remaining wild-type alleles using homology sequences from the previously modified allele. Eventually, all of the wild-type alleles are converted into modified alleles. This strategy allows self-propagation of the desired trait, super-Mendelian inheritance of a transgene, and genetic modifications of specific populations or entire species.

Another way to enrich biallelic edited cells is by using a universal surrogate reporter system (HDR-USR)^66^. Compared with the surrogate reporter-integrated donor system^61^, the HDR-USR system functions by itself in an episomal manner^66^. Because the HDR-USR surrogate vector is not integrated into the genome, it allows scarless genome editing without introducing insertional mutagenesis and unwanted exogenous sequences to the genome. Therefore, this HDR-USR system is particularly useful for knock-in of non-coding variants to study functional regulatory elements. It also can be employed to knock-in protein-coding variants in exons. The HDR-USR system contains a truncated puromycin-resistant gene (Figure 1E). Puromycin-resistant function is restored through self-cleavage from expressed Cas9/sgRNA complex targeting truncated puromycin-resistant gene and self-repair via HDR using the full-length puromycin-resistant gene as a homologous intra-molecular template. Edited cells that have undergone HDR can be selected and enriched by co-transfecting this HDR-USR surrogate vector with the sgRNA expression cassette and integration of donor cassette into the cells. Compared with the commonly used surrogate reporter plasmids (for example, fluorescent proteins and antibiotic-resistant genes) that are used for the enrichment of transfection-positive cells, this HDR-USR surrogate vector displays better performance for the enrichment of HDR-edited cells.

It was recently shown that 5′ modification to the PCR-generated long dsDNA donors (~400 bp) strongly enhances HDR events (Figure 1F)^67^. Importantly, 5′ modification of the donors can tackle the problem associated with the integration of multimers due to joining together the multiple templates end to end, thereby enabling only one whole copy of the desired DNA to be inserted into the genome. This strategy also enables DNA to be edited in a more controlled way and promotes precise gene knock-in. Cas9 RNP complexes and 5′-modified linear double-stranded DNA donors with short homology arms (~50 bp) have also successfully been used to enable efficient gene knock-in^68^. This 5′-end modification of donor does not require long homology arms (for example, plasmid donor templates) and helps to reduce the background signal from the DNA repair templates. Modulation of targeting vectors to provide 3′ overhangs at both ends could also enhance HDR frequency by facilitating the assembly of proteins involved in strand invasion and new DNA synthesis in HDR^69^.

Gene knock-in and gene correction efficiency can be increased by enhancing HDR efficiency. HDR events are increased by synchronizing and enriching the cells in the G_2_/M cell cycle phase^70^. Alternatively, Cas9 expression is synchronized in S/G_2_/M by controlled timing of CRISPR/Cas9 RNP complex delivery^71^ or using a fusion of Cas9 and the N-terminal region of human Geminin^25^. HDR has also been successfully activated in quiescent stem cells and primary cells through controlled cycling and quiescence^72^. In this case, these quiescent cells are induced to briefly enter the cell cycle for HDR-based gene editing using the timed administration of a small-molecule cocktail (for example, SCF, TPO, and Flt3L). After gene editing, these HDR-edited cells are induced back into quiescence using another combination of small molecules (for example, rapamycin and CHIR99021). A modified version of Cas9 has also been employed to increase the HDR/NHEJ ratio by limiting the Cas9 nuclease activity in the G_1_ phase of the cell cycle^73^. HDR efficiency has also been improved by using small molecules that directly modulate HDR pathways, such as RS-1 (RAD51 activator)^74,75^, L755507 (β3-adrenergic receptor agonist)^76^, and nocodazole (a G_2_/M phase synchronizer)^27^. Ectopic co-expression of RAD52 and dn53BP1 could improve HDR efficacy without altering Cas9 off-target activity^77^. In addition, overexpression of an engineered RAD18 variant (e18) promotes HDR by suppressing the localization of the NHEJ-promoting factor 53BP1 to double-strand breaks^78^. To further promote HDR events, small molecules are used to inhibit the NHEJ activity by disrupting a key enzyme in the NHEJ pathway, such as DNA ligase IV^79^, KU70^80^, and 53BP1^81^. In addition, attenuation of histone deacetylase 1 (HDAC1) and HDAC2 activities was recently shown to facilitate Cas9 access and binding to the targeted DNA, thereby enhancing HDR events and gene knock-in efficiency^82^. As such, a desired editing outcome can be genetically manipulated or chemically induced by altering the choice of DNA double-strand break repair to favor a specific DNA repair pathway^72,83^. For example, small molecules (chemical compound) and factors (genes) identified in high-throughput screens can be used to enhance or inhibit a specific DNA repair pathway for intended genome editing. To realize such applications, many other unknown small molecules and factors that potentially target the DNA damage response can be screened using a recently developed scalable CRISPR/Cas9-based fluorescent reporter assay to assess their influence on DNA double-strand break repair choice^84^. The underlying mechanisms by which these small molecules and factors regulate double-strand break repair then can be evaluated.

However, the use of small molecules or disruption of NHEJ and histone modifier genes can cause genome-wide changes in gene regulation and expression. Therefore, Cas9 was recently fused to CtIP, a key protein in early steps of homologous recombination, to stimulate precise transgene integration by HDR^85^. Directing CtIP-mediated homologous recombination to the CRISPR/Cas9 cutting site can minimize a global effect following DNA repair. On the other hand, fusions of Cas9 to DN1S (dominant-negative mutant of 53BP1) enhance HDR and precise gene knock-in by inhibiting NHEJ events specifically at Cas9 cleavage sites^86^. The use of this Cas9-DN1S fusion protein can circumvent the issue associated with the unwanted effects of global NHEJ inhibition. Another strategy to improve HDR efficiency is to covalently link the DNA repair templates to CRISPR/Cas9 complexes^87–89^. For example, to shuttle the HDR template to the nucleus, truncated Cas9 target sequences (16 bp) are added to the ends of the template for interacting with Cas9 RNPs (Figure 1G)^90^. Because Cas9 contains nuclear localization sequences (NLSs), Cas9 RNPs along with the associated HDR template can be transported together into the cell nucleus. To further enhance HDR efficiency, multiple copies of DNA repair templates are conjugated to multiple sites on a Cas9 via the conjugation adaptor^89^. Each conjugation adaptor on Cas9 bears the complementary sequence to the DNA repair template and thus multiple conjugation adaptors enable multivalent display of DNA repair template on Cas9. These conjugation strategies ensure the uptake and co-localization of both repair template and Cas9–sgRNA RNP complex in an individual cell^87,88^.

Machine learning has been used to further improve HDR efficiency by designing the optimal targeting strategy and repair template^91^. Deep learning has helped to predict editing products and genotypes such as on-target mutagenesis and off-target activities upon CRISPR-mediated gene correction of pathogenic variants in human cells^92–96^. In addition to aid designing an optimal DNA sequence, deep learning can also improve editing activity of CRISPR by taking into account chromatin accessibility and epigenetic features^97,98^.

### Synthesis-dependent strand annealing (SDSA)-based approaches

ssODN^30^ and linear double-stranded DNA PCR fragments^31^ enable efficient gene knock-in via a synthesis-dependent strand annealing (SDSA)-like DNA repair mechanism. Compared with HDR-mediated knock-in using dsDNA plasmid template (>500 bp each homology arm), ssODN (50–80 bp each homology arm) and PCR fragment (~35 bp homology arms) provide higher efficiency for knock-in of small DNA modifications (for example, SNP)^30,31^. Given their high efficiency of a point mutation knock-in, a fluorescent tag is not required for the PCR fragment or ssODN donor. Several mechanistic models have been proposed to explain how a point mutation is introduced to the genome through DNA repair machinery mediated by a short single-stranded DNA oligonucleotide and CRISPR/Cas9 system. Two promising models are excision and corrective therapy (EXACT)^99^ and single-stranded template repair (SSTR)^100^ models. EXACT uses ssODNs to serve as both a bandage and a template for gene correction, whereas SSTR uses the Fanconi anemia DNA repair pathway for targeted mutagenesis.

The mechanism of gene repair directed by the ssODNs was elucidated in detail well before the CRISPR era^101–105^. In the case of single-agent gene editing, point mutations and base lesions are repaired by ssODNs in the absence of the CRISPR RNP complex^106^. This ssODN-mediated DNA repair machinery can be enhanced by stalling of replication forks and synchronizing the cells in the early S cell cycle phase during ssODN exposure^106,107^ or using anticancer drugs to induce double-strand DNA breakage^108,109^. Subsequently, CRISPR/Cas9 RNP complex has been used to improve point mutation repair directed by a short ssODN through induction of double-stranded breaks at the target genomic loci^99^. Nevertheless, the mechanism of action of DNA repair directed by an ssODN as the sole gene-editing agent is generally similar to that of ssODN and CRISPR/Cas9 working in concert. Interestingly, single-nick-induced gene editing using ssODN and Cas9n generates conversion tracts biased either mostly unidirectional or bidirectional depending on the relative strandedness of the ssODN and the nick^110^. In contrast, the unidirectional conversion pathway is preferentially used upon CRISPR/Cas9-induced double-strand DNA breaks. A strand bias in ssODN-mediated gene editing could be due to different accessibility of the DNA strands during DNA replication, irrespective of transcriptional status^111^. The sequences surrounding the targeted nucleotide also influence strand bias, while occupancy by transcriptional complexes alone does not dictate strand bias^112^. Notably, unmodified ssODNs are the most efficient in gene correction, while the addition of chemical modifications to the ssODN did not further improve its efficacy^111^.

**Figure 2.**
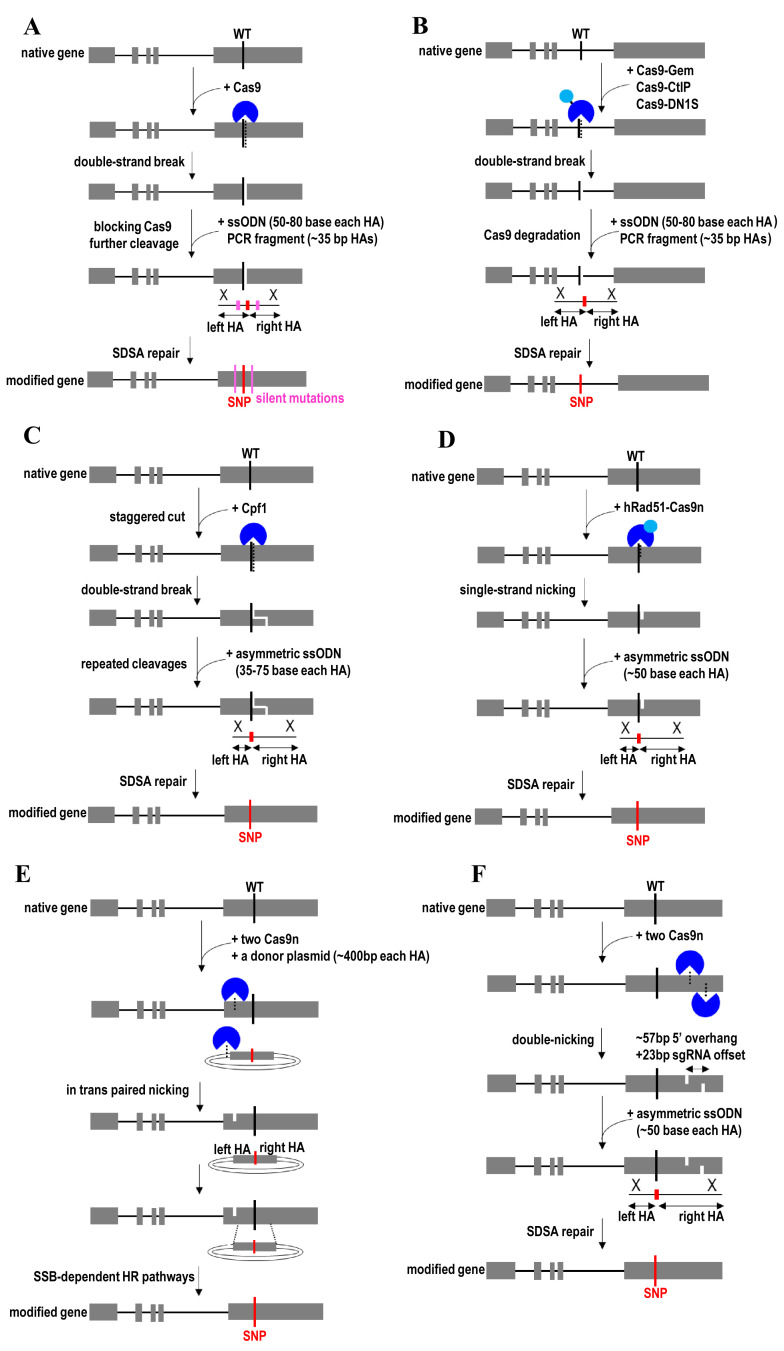
Synthesis-dependent strand annealing (SDSA)-mediated gene knock-in and gene correction strategies. (**A**) Exonic SNP knock-in using CRISPR/Cas9 along with a short single-stranded donor oligonucleotide (ssODN) or a linear dsDNA PCR fragments. (**B**) Intronic SNP knock-in using Cas9-Gem, Cas9-CtIP, or Cas9-DN1S along with a short ssODN or a linear dsDNA PCR fragment. (**C**) Exonic SNP knock-in using CRISPR/Cpf1 and asymmetric donor ssODN. (**D**) Exonic SNP knock-in using hRad51-Cas9 nickase and asymmetric donor ssODN. (**E**) Exonic SNP knock-in by in trans paired nicking to target genomic DNA and donor plasmid using a pair of Cas9 nickases. (**F**) Exonic SNP knock-in by simultaneous nicking both DNA strands using a Cas9 nickase mutant with a pair of sgRNAs targeting opposite DNA strands. Cas9n, Cas9 nickase; CtIP, a key protein in early steps of homologous recombination; DN1S, dominant-negative mutant of 53BP1; Gem, human Geminin protein; HA, homology arm; HR, homologous recombination; hRad51, human RAD51 recombinase; SNP, single-nucleotide polymorphism; SSB, single-stranded break.

Silent mutations are usually introduced into the PAM sequence or the sgRNA seed sequence of the donor template to block further Cas9 targeting and recutting after undergoing HDR (Figure 2A). This can prevent unwanted mutations (for example, indels) introduced into the DNA upon subsequent NHEJ repair of Cas9-induced double-strand breaks. However, the insertion of silent mutations is not advisable when designing CRISPR/Cas9 to target the non-coding regions for knock-in of non-coding variants into the genome. In this case, the use of Cas9-Gem allows indel-free knock-in at the target locus by facilitating the degradation of Cas9 nuclease during the G_1_ phase of the cell cycle (Figure 2B)^113^. Gem is derived from human Geminin protein that is highly expressing during the S and G_2_ phases. Therefore, the fusion of Gem to Cas9 allows Cas9 to persist only during S and G_2_ phases. Gene correction efficiency is further increased by using CRISPR/Cpf1^114^ and asymmetric donor ssODN^115^ to enhance HDR (Figure 2C). Because the Cpf1 cleavage site is distal from the PAM sequence and sgRNA recognition site, Cpf1 enabled higher HDR rates than Cas9 by allowing repeated cleavages before indel mutations terminate targeting^114^. Compared with the blunt ends produced by Cas9, Cpf1 enhances gene insertion and knock-in by generating sticky ends upon DNA cleavage via a staggered DNA double-stranded break^116,117^. Cpf1-derived synthetic chimeric nucleases provide a broader targeting scope and a higher editing specificity than Cpf1^118^. Meanwhile, asymmetric donor DNA enhances gene insertion and replacement by biasing the choice of DNA repair pathway toward SDSA or increasing the local concentration of the donor^115^. It is also possible to use a synthetic long ssODN (1–3 kb) donor bearing two homology arms (60–300 bp each) along with CRISPR/Cas9 to integrate a long transgene into the genome^119^.

Because imprecise repair of Cas9-induced double-stranded DNA breaks can give rise to unpredictable deleterious mutations and complex chromosomal rearrangements^120,121^, Cas9n mutant is used to nick single-stranded DNA for seamless genome editing^29^. DNA nicks are repaired by the alternative HDR pathway (for example, SDSA pathway), especially when canonical RAD51/BRCA2-dependent HDR is compromised or down-regulated^122^. Studies have shown that Cas9 D10A (a D10A mutation on RuvC catalytic domain) is more potent in mediating HDR than Cas9 H840A (an H840A mutation on HNH catalytic domain)^29^. For example, Cas9n is fused to hRad51 mutants to improve HDR efficiency while minimizing undesired consequences of double-stranded DNA breaks and off-target mutagenesis (Figure 2D)^123^. This strategy uses ssODN as donor templates and does not require the inclusion of PAM-blocking silent mutations to prevent target recutting by Cas9n after undergoing SDSA. hRad51 participates in the repair of nicked DNA via strand invasion, and the nick-mediated HDR efficiency is improved using hRad51 mutant forms to disable its binding to BRCA2 and self-associate. Therefore, localizing hRad51 mutants to a targeted DNA nick can increase the frequency of HDR in response to a DNA nick.

In trans paired nicking was another recently developed strategy to enable efficient seamless genome editing without inducing double-stranded DNA breaks^124–126^. This strategy uses a pair of Cas9ns to generate coordinated single-strand breaks in donor plasmids and chromosomal target sites (Figure 2E). Recombination between nicked plasmid donor and a nicked target sequence then proceeds through SSB-dependent HR pathways. This strategy helps to avoid mutagenizing unmodified alleles and minimize large-scale chromosomal rearrangements, thereby enabling accurate gene knock-in, and maintains target protein dosages^125^. It also allows the editing of genes that are essential for cell function and survival. As an alternative strategy, a Cas9n mutant with a pair of sgRNAs targeting opposite DNA strands nearby (40–70 bp apart between two nicking sites) is used to induce a staggered double-strand DNA breaks with overhangs at the target locus by nicking both DNA strands simultaneously (Figure 2F)^29,127^. The PAM site for each Cas9n should face the outside of the target region to enable robust gene editing. While maintaining high on-target efficiencies, this double nicking strategy enhances genome-editing specificity by reducing off-target activity. Although both Cas9n must function in concert to make a double-strand break, paired Cas9n is sometimes more efficient than individual Cas9 nuclease for gene disruption^128^.

### Microhomology-mediated end joining-based approaches

**Figure 3.**
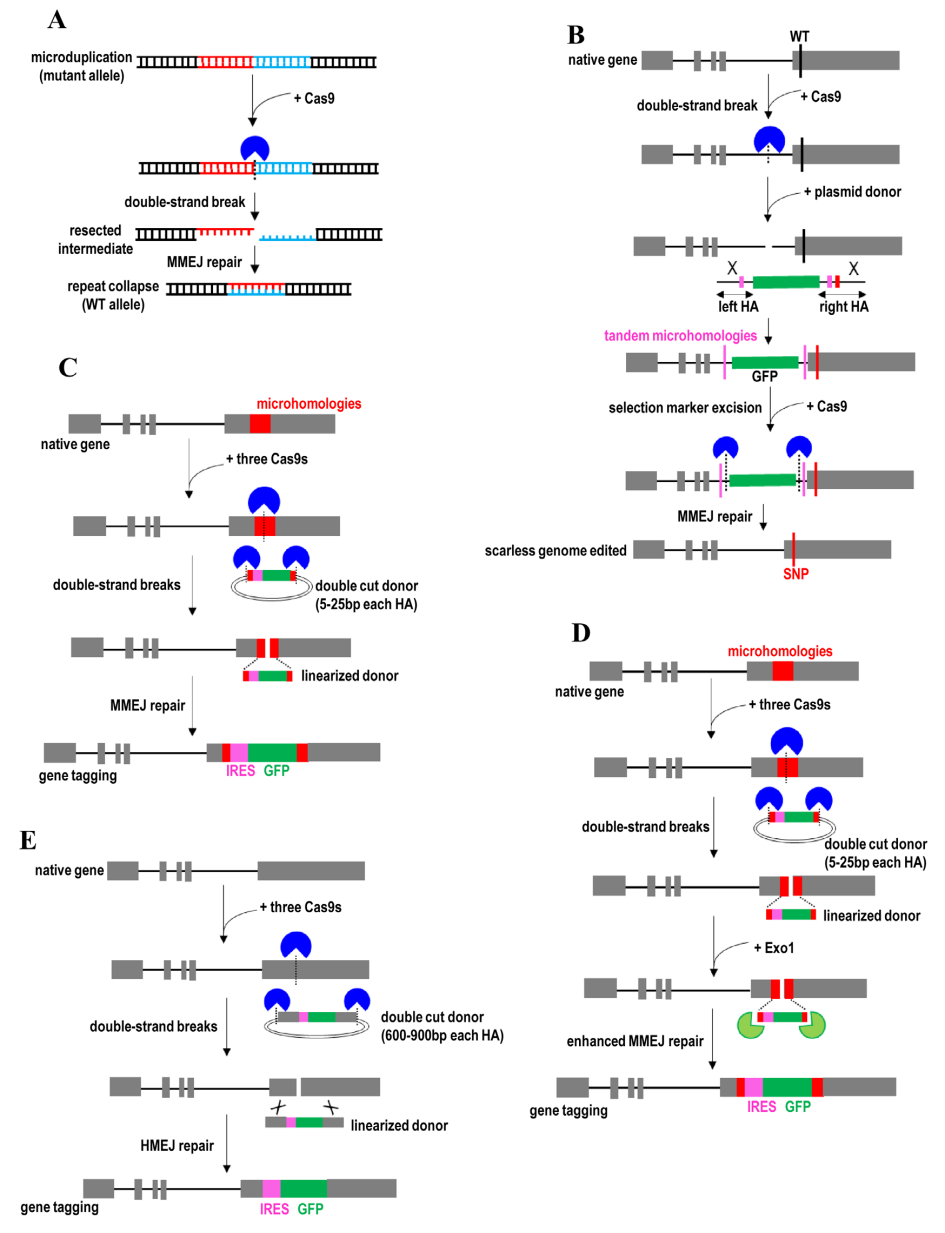
Microhomology-mediated end joining (MMEJ) and homology-mediated end joining (HMEJ)-mediated gene knock-in and gene correction strategies. (**A**) Gene correction of microduplications by CRISPR/Cas9-mediated DNA double-stranded break near the center of the duplication. (**B**) Exonic SNP knock-in by CRISPR/Cas9-mediated integration of a selection marker and two short tandem microhomologies at intron and an SNP at exon, followed by removal of the selection marker using two CRISPR/Cas9s targeting the region between the selection marker and tandem microhomologies. (**C**) Transgene knock-in using a double-cut donor plasmid with short microhomologies and Cas9 with three different sgRNAs. (**D**) Transgene knock-in facilitated by exonuclease 1 (Exo1). (**E**) Transgene knock-in using a double-cut donor plasmid with long homology arms. GFP, green fluorescent protein; HA, homology arm; IRES, internal ribosome entry site; SNP, single-nucleotide polymorphism; WT, wild-type.

Another DNA repair pathway, namely MMEJ, has also been exploited for efficient and precise gene correction^35^. MMEJ pathway can be a promising alternative to classic HDR because HDR is inefficient in many cell types. MMEJ is active during most periods of the cell cycle, whereas the activity of HDR is restricted to the S/G_2_ phases. MMEJ uses extremely short microhomologous sequences (5–25 bp) to align the broken strands before joining^129^. For example, CRISPR/Cas9 is employed to induce a DNA double-stranded break near the center of a disease-causing microduplication (Figure 3A)^35^. This microduplication then is reverted to the wild-type genomic sequence upon MMEJ repair of double-stranded breaks. The wild-type genomic sequence is no longer targeted by the sgRNA that was used to target the microduplication, thereby avoiding another round of cleavage by Cas9. This strategy enables efficient and precise gene replacement without using exogenous DNA donors and can be used to edit a wide range of microduplication lengths. However, the application of this MMEJ-based approach is limited to this class of pathogenic mutations.

MMEJ-based strategy has also been recruited for scarless genome editing^36^. In this case, a plasmid donor with two overlapped homology arms bearing two short tandem microhomologies is used (Figure 3B). These two homology arms are flanking a selection marker, while a point mutation is juxtaposed with this selection marker. CRISPR/Cas9 is first used to induce a DNA double-stranded break in the intronic region proximal to the exonic SNP site. These two engineered microhomologies, a point mutation, and the selection marker in the donor then are integrated at the CRISPR/Cas9 cleavage site. Cas9 with two different sgRNAs then is used to cleave the DNA sequences between the selection marker and engineered microhomologies. MMEJ repair pathway will be stimulated to use these two engineered microhomologies to align the broken strands before joining. This leads to the deletion of one copy of tandem homology along with intervening selection marker sequences, leaving behind only the desired point mutation at the locus. The removal of sgRNA target sites and PAM sequences enables the protection of corrected and mutant alleles from subsequent cleavage by Cas9. This strategy is useful for the scarless excision of a selectable marker and biallelic modifications.

In addition to gene correction, the MMEJ pathway has been exploited to insert large DNA fragments (for example, tags) into the genome^130,131^. In this case, Cas9 with three different sgRNAs is co-expressed in the cell (Figure 3C). One sgRNA targets the genomic site and two sgRNAs target the 5′ and 3′ ends of the donor sequence. One sgRNA is designed to target the center of microhomologous sequences (~20 bp) in the genome. The microhomologous sequences and exogenous DNA sequences (for example, selection marker) in the donor are flanked by two sgRNA target sites. CRISPR/Cas9 then is used to linearize donor plasmid and release the exogenous DNA sequence with microhomologous sequences. This double-cut donor vector provides a higher gene knock-in efficiency than the conventional circular donor plasmid. Because the double-cut donor plasmid contains microhomologous DNA ends corresponding to the genomic cleavage site, a copy of tandem homology along with intervening exogenous DNA sequences is integrated into the genome upon MMEJ repair. This MMEJ-based approach uses only very short microhomologies and enables flexible gene knock-in without introducing unwanted exogenous sequences (for example, vector backbone sequence)^130,131^. Exonuclease 1 (Exo1) was also recently used to enhance MMEJ-mediated knock-in^132^. Exo1 facilitates end resection of double-strand breaks at both the genomic locus and the donor vector, thereby promoting the alignment between cleavage genomic site and linearized donor using microhomology (Figure 3D).

### Homology-mediated end joining (HMEJ)-based approaches

The MMEJ-based strategy was subsequently devised to an HMEJ approach by enabling more efficient targeted transgene integration using longer and more stable homology arms^27,37–39^. In this case, CRISPR/Cas9 is designed to cleave both the targeted genomic locus and transgene donor vector that contains long homology arms (600–900 bp each homology arm) (Figure 3E). This HMEJ-based strategy provides a higher editing efficiency and better fidelity than MMEJ, particularly in non-dividing cells and adult animals^37^. HMEJ-based strategy also allows efficient knock-in of multiple genes without undergoing selection and enriching processes^38^. HMEJ efficiency is further improved by combining the use of this double-cut donor vector with the cell cycle regulators (for example, Nocodazole and CCND1)^27^. Targeted transgene integration mediated by HMEJ would not alter global gene expression profiles^39^. Moreover, targeted integration of a large gene payload is useful for the creation of reporter cells, production of recombinant pharmaceutical proteins, and efficient biallelic gene disruption. However, similar to MMEJ-based knock-in approaches, HMEJ requires a PAM sequence close to the CRISPR/Cas9 cleavage site in the genome.

## Homology-independent gene knock-in and gene correction strategies

### Non-homologous end joining (NHEJ)-based approaches

**Figure 4.**
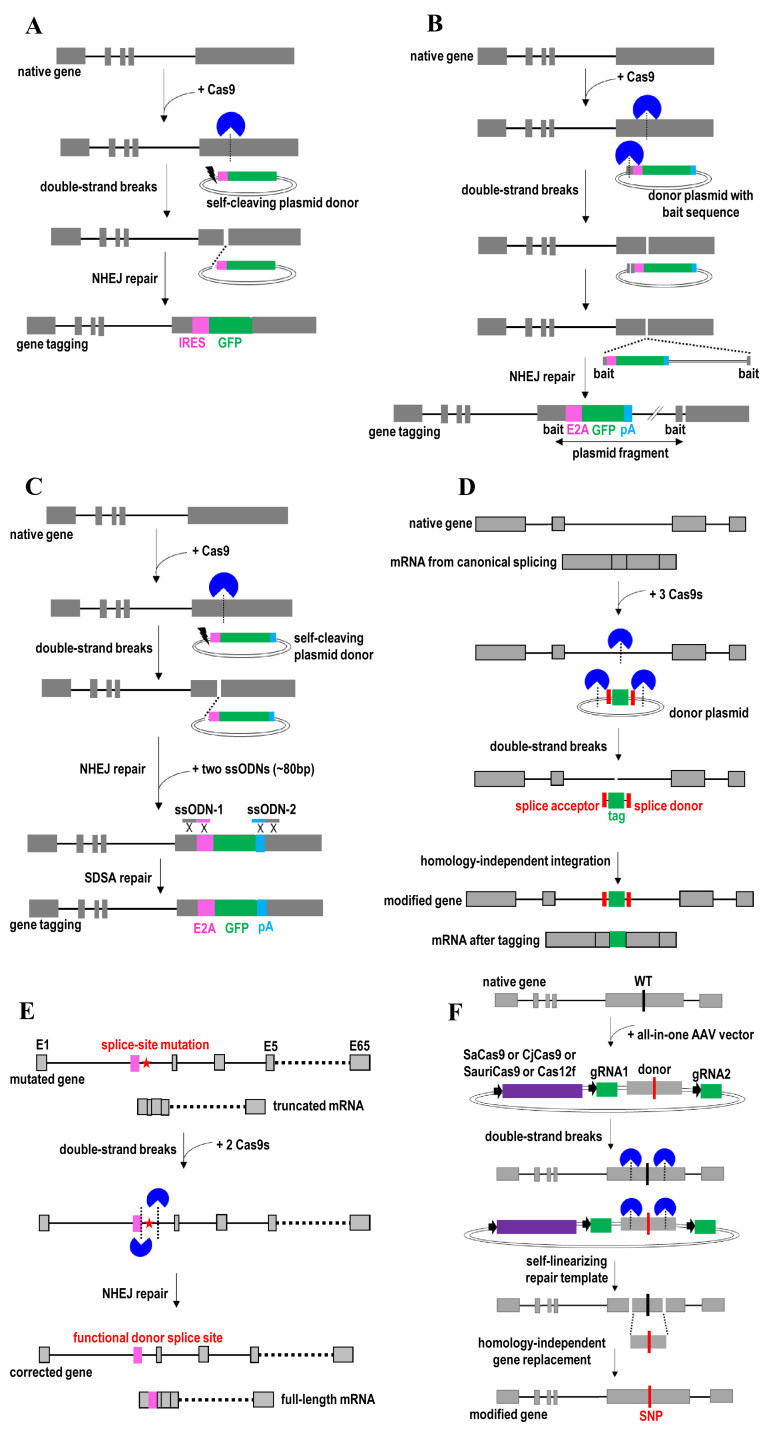
Non-homologous end joining (NHEJ)-mediated gene knock-in and gene correction strategies. (**A**) Transgene knock-in by introducing double-strand DNA breaks to both genome and single-cut donor. (**B**) Large transgene knock-in by including the bait sequence onto a donor plasmid. (**C**) Very large transgene knock-in using CRISPR/Cas9 and single-cut donor along with two additional short ssODNs. (**D**) Gene tagging by CRISPR/Cas9-mediated integration of a tag flanked by splice acceptor and donor sites. (**E**) Restore the function of donor splice site and full-length mRNA by CRISPR/Cas9-mediated deletion of the intervening intronic sequence that contains a pathogenic splice-site mutation. (**F**) Gene replacement using self-linearizing all-in-one AAV repair templates and CRISPR/Cas9s to generate two simultaneous double-strand breaks in both genomic DNA and repair template. AAV, adeno-associated virus; GFP, green fluorescent protein; IRES, internal ribosome entry site; SDSA, synthesis-dependent strand annealing; ssODN, single-stranded donor oligonucleotide.

NHEJ repair occurs throughout the cell cycle, whereas the HDR event is restricted to S/G_2_ phase in dividing cells^133^. Importantly, NHEJ is a predominant DNA repair pathway in non-dividing cells and post-mitotic tissues (for example, neurons and human organoids). Therefore, the efficiency of insertional mutagenesis and gene correction is limited by NHEJ that competes with HDR. In fact, NHEJ-based knock-in was recently shown to have a higher efficiency than HDR-mediated gene targeting^9,134,135^. In this case, double-strand DNA breaks are introduced to both the genome and donor template for mediating transgene insertion via the NHEJ repair pathway (Figure 4A)^40,135^. The donor plasmid is linearized using Cas9 to cleave one sgRNA target site (for the single-cut donor) or two sgRNA target sites at both sides of the transgene (for the double-cut donor). However, double-cut donor is less efficient than the single-cut donor because the former will generate two DNA fragments that compete for genomic integration^134^. The linearized donor plasmid then is directly ligated to the broken genomic DNA ends upon NHEJ repair. NHEJ efficiently re-ligates DNA ends without mistakes and it does not require regions of homology for precise transgene insertion. However, the donor can be inserted in either orientation of the broken genomic DNA ends upon NHEJ repair. Off-target DNA double-strand breaks would also lead to random donor insertion to the unintended genomic sites. To circumvent this issue, a short homology DNA sequence bearing the Cas9 target sequence (bait sequence) is introduced onto a donor plasmid (Figure 4B)^136^. In this case, concurrent cleavage of the target genomic locus and bait plasmid sequence leads to efficient targeted integration of a large transgene via NHEJ pathway. Nevertheless, the insertion is independent from the homology sequence between the target locus and the bait in the donor plasmid. Similar NHEJ strategy can also be achieved by using Cpf1 to create sticky ends at the DNA cleavage site^117^.

To replace large genomic regions (for example, 58 kb) or integrate a very large transgene (for example, 200 kb) into the genome, two additional short ssODNs (~80 bp) are introduced together with self-cleaving plasmid donor and CRISPR/Cas9 targeting the genomic locus into the cell (Figure 4C)^119^. Upon double-strand breaks, these two ssODNs ligate each cut end to join the genomic DNA and the plasmid donor via the SDSA repair pathway. This integrated approach based on NHEJ and SDSA is useful for protein tagging, labeling of cellular structures, generating reporter lines, creating loss-of-function alleles, and lineage-tracing experiments^119,134,135^. It eliminates the need of attaching homology arms to the donor vector. It is also more efficient than traditional HDR-based knock-in strategies to assess and edit the silenced genomic loci or compact chromatin areas^134^.

The NHEJ-based knock-in strategy has also been employed to program exon skipping and gene tagging by modulation of gene splicing^137^. Gene splicing is manipulated by mutating specific DNA bases within splice acceptor sites or the addition of splice acceptor and donor sites to the donor DNA. For example, to add a tag to the endogenous mRNA, a donor plasmid with a tag flanked by splice acceptor and donor sites is used (Figure 4D)^137^. Two sgRNA target sites are located at both ends of the tag with splicing sites. CRISPR/Cas9 is first used to generate double-strand breaks in both the intron and donor. A tag together with splicing sites in this donor then is introduced to the intronic region via a homology-independent integration pathway. Through alternative splicing, the tag will be added to the coding sequence upon mRNA expression. Similarly, exon inclusion can be achieved by replacing the tag with a synthetic exon. Gene tagging is useful for interrogation of protein function, localization, and interactions in their native context within living cells. Therefore, it can avoid artifacts associated with overexpression of exogenous transgenes. Meanwhile, exon inclusion is useful to derive a protein with a new function and property. Actually, a similar strategy has been previously employed to tag at the C and N terminus of an exon using a generic donor plasmid^138,139^. This NHEJ-based gene tagging approach is scalable for tagging endogenous loci because of the use of the universal donor DNA. However, compared with the N- and C-terminal tagging approach, this intron-tagging strategy can prevent the disruptive or deleterious impact on the coding sequence. Another efficient way to tag the endogenous genes is by mutating specific DNA bases within splice acceptor sites using CRISPR technology^41^. For example, a pathogenic splice-site mutation causes the exclusion of exon 2 from an mRNA and generates the truncated protein (Figure 4E). CRISPR/Cas9 is used to excise an intronic region containing this mutation^41^. Deletion of this mutation will restore the function of donor splice site through NHEJ repair. The edited gene can now produce the full-length mRNA and restore the protein function. This strategy is useful for correcting non-coding mutations and creating a functional splice donor site by deleting the intervening intronic sequence that contains a splice-site mutation.

An all-in-one AAV vector with two sgRNAs, SaCas9^140,141^ (or other small Cas variants such as CjCas9^142^, SauriCas9^143^, Cas12f^144^, St1Cas9^145^, and CRISPR-CasPhi^146^), and a self-linearizing repair template was recently used for gene replacement (Figure 4F). In this case, CRISPR/Cas9 is first used to generate two simultaneous double-strand breaks in both genomic DNA and repair template. This leads to the replacement of the intervening sequence in genomic DNA with a repair template containing the desired mutation or wild-type sequence via a homology-independent DNA repair pathway. An integrated use of single AAV vector platform and homology-independent gene replacement approach enables robust gene correction of pathogenic variants *in vivo*. The use of this all-in-one AAV vector enables delivery of CRISPR/Cas9 and repair template using only a single AAV vector, thereby simplifying AAV production and improving delivery efficiency and CRISPR editing potency^140^.

## Other gene knock-in and gene correction strategies

### Base editing

Because the imprecise repairing of Cas9-induced DNA double-stranded breaks can lead to on-target mutagenesis and chromosomal rearrangements^147^, base editing provides a safer way to perform gene correction. Base editor, a CRISPR-based synthetic biology tool, was recently repurposed to enable programmable editing of a target base in genomic DNA by harnessing the base excision repair (BER) ability^42,43^. BER accurately repairs base lesions and single-strand breaks throughout the cell cycle^148^. Base editor is favorable to Cas9 because it enables clean mutational knock-in by direct conversion of nucleotides in genomic DNA without inducing double-strand DNA breaks that can generate unwanted indel mutations. Nevertheless, designing multiple guide sequences of base editor to target several genomic sites in trans may still be able to lead to double-strand DNA breaks and result in the deletion of the intervening segment between two sgRNA target sites. Importantly, base editing is particularly useful for manipulating point mutations and correcting pathogenic variants in postmitotic cells and most of the somatic tissue *in vivo*^149–152^. Nucleotide substitutions with base editor generated much excitation partly because the HDR event is highly inefficient in non-dividing cells and the NHEJ-based editing can lead to on-target indel formation. The use of base editors also can simplify the delivery of editing machinery because no homologous DNA repair template is required to edit the genomic DNA sequence.

**Figure 5.**
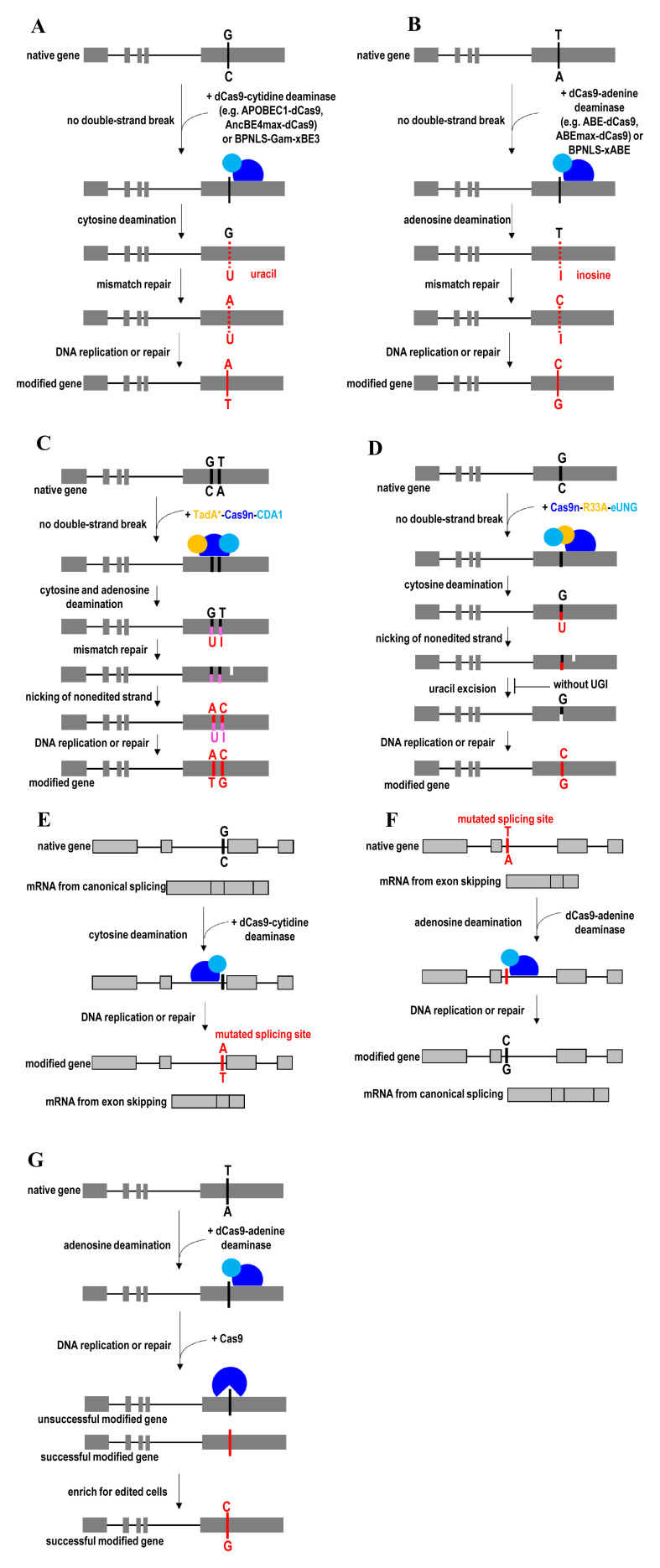
Base excision repair (BER)-mediated gene knock-in and gene correction strategies. (**A**) C-to-T (or G-to-A) substitution by direct conversion of cytidine to uridine using cytidine base editors. (**B**) A-to-G (or T-to-C) substitution by direct conversion of adenine to inosine using adenine base editors. (**C**) Concurrent adenine and cytosine editing by a dual-deaminase CRISPR base editor. (**D**) Targeted C-to-G base transversions by a Cas9n-R33A-eUNG base editor. (**E**) Program exon skipping and (**F**) restore full-length mRNA by mutating target DNA bases within splice acceptor sites. (**G**) Enrich base-edited cells by eradicating non-edited cells using an inducible active Cas9 with the same sgRNA as the base editor. ABE, adenine base editor; APOBEC1, apolipoprotein B mRNA-editing enzyme, catalytic polypeptide 1; BPNLS, biparticle nucleus localization signal; eUNG, an *Escherichia coli*–derived uracil DNA N-glycosylase; R33A, a rat APOBEC1 cytidine deaminase variant; TadA, adenosine deaminase; UGI, uracil glycosylase inhibitor.

In general, base editors are classified into two broad classes: cytidine base editors and adenine base editors. Cytidine base editor (for example, APOBEC1-dCas9) mediates the direct conversion of cytidine to uridine, which eventually leads to a C-to-T (or G-to-A) substitution (Figure 5A)^42^. Meanwhile, adenine base editor (for example, ABE-dCas9) mediates the direct conversion of adenine to inosine, which eventually leads to an A-to-G (or T-to-C) substitution (Figure 5B)^43^. Concurrent adenine and cytosine editing has also been achieved by using a dual-deaminase CRISPR base editor^153^ or an engineered TadA* enzyme of adenine base editors^154^. The dual-deaminase CRISPR base editor can concurrently introduce A-to-G and C-to-T substitutions to the target DNA in the genome, thereby enabling creation or correction of multi-nucleotide variants (Figure 5C). In addition to DNA base transitions (pyrimidine-to-pyrimidine or purine-to-purine conversions), targeted C-to-G base transversions have been achieved by using a Cas9n-R33A-eUNG^155^. This C-to-G base editor consists of a Cas9n, an *Escherichia coli*–derived uracil DNA N-glycosylase (eUNG), and a rat APOBEC1 cytidine deaminase variant (R33A) (Figure 5D). The specificity of the C-to-G base editor was improved by removing the eUNG domain from it to yield Cas9n-R33A. C-to-G editing permits nucleotide substitutions and mutational correction at the desired genomic sites not possible with cytidine and adenine base editors. Compared with transition mutations, introduction of transversion mutations into the transcription factor binding sites may exert stronger effects on transcription factor binding and gene expression.

Base editors have also successfully been used to program exon skipping^156^ (Figure 5E) and restore full-length mRNA^157^ (Figure 5F) by modulation of gene splicing through mutating target DNA bases within splice acceptor sites. Base conversion efficiency mediated by both cytidine^42^ and adenine^43^ base editors is the highest when the target cytidine or adenine is at protospacer position 5. Nevertheless, the base editing can occur within a window of about 5 nucleotides (at protospacer positions 4–8). To broaden the targeting scope, the editing window of base editors was recently expanded from 4 or 5 nucleotides to 8 or 9 nucleotides by circularly permuting the Cas9n domain of base editors^158^. To narrow the editing window for single-nucleotide replacement at a specific position, high-precision base editors were engineered by optimizing the length of the linker between the deaminase domain and the Cas domain of the editor^159,160^.

Although the base editors enable direct conversion of C-to-G base transversions and all four transition mutations (C to T, A to G, T to C, and G to A) at target loci in living cells, base editing encounters several limitations. The application of base editing is restricted to point mutation substitutions. Base editing also has a limited targeting scope, low editing efficiency, and high off-target activities^161–163^. It is a concern that adenine base editors can display unexpected on-target cytosine deamination activity by converting cytosine to guanine or thymine^154^. Base editors also can induce unwanted bystander nucleotide conversions when repeated sequences (for example, multiple Cs) are present in the editing window. Therefore, several next-generation base editors were recently engineered to address these issues. These next-generation base editors introduce desired point mutations at a target locus at significantly higher efficiency and fewer undesired by-products than a CRISPR/Cas9-mediated HDR approach^42,43^. The genome-targeting scope of these next-generation base editors is also broader than the dCas9- or Cas9n-derived base editor^164^.

Next-generation cytidine base editors enhance desired base editing and minimized on-target indel formation by fusing uracil glycosylase inhibitor to the base editor (for example, APOBEC1-dCas9-UGI) and employing a Cas9n (for example, APOBEC1-Cas9n-UGI) targeting the non-edited strand^42^. Uracil glycosylase inhibitor (UGI) blocks uracil DNA glycosylase from reversing the U:G pair to a C:G pair, thereby improving base-editing efficiency. Replacing dCas9 with Cas9n improves base-editing efficiency by inducing mismatch repair pathway to resolve the U:G mismatch into desired U:A and T:A products. To stimulate mismatch repair, Cas9n is used to nick the non-edited strand containing a G opposite the edited U. Later, APOBEC1-dCas9-UGI and APOBEC1-Cas9n-UGI were further evolved into BE4max or AncBE4max by improving nuclear localization and optimizing codon usage for maximum expression of base editors^44^. The BE4max and AncBE4max provide higher base-editing efficiency than the older cytidine base editors. Nevertheless, the editing window remains similar to the previously established cytidine base editors. With the phage-assisted continuous evolution of the base-editor method, these cytosine base editors were subsequently evolved into evoAPOBEC1-BE4max, evoFERNY-BE4max, and evoCDA1-BE4max^165^. Compared with BE4max and AncBE4max, these three evolved base editors are more efficient at editing cytosine in the GC context. EvoFERNY is also smaller than APOBEC1, thereby enabling the packaging of EvoFERNY-BE4max into the AAV vector for *in vivo* applications. EvoCDA1-BE4max is useful for a difficult-to-edit site or cell type. It should be noted that their editing windows and targeting scope are different from the original BE4max. Human APOBEC3G-Cas9n fusion was another recently engineered cytidine base editor to reduce unwanted bystander activities and improve precision of base editing in the CC context (repeated Cs)^166^. To minimize unwanted RNA-editing activity without sacrificing on-target DNA-editing activity, Cas9n-UGI was fused to non-APOBEC1 cytidine deaminases, such as an enhanced human APOBEC3A (eA3A) and human activation-induced cytidine deaminase (hAID)^167^. These base-editor variants also did not self-edit their own transcripts, thereby avoiding heterogeneity in base-editor coding sequences.

Similar to cytidine base editor, next-generation adenine base editor (for example, ABE7.10) was engineered to mediate the conversion of A/T to G/C in genomic DNA^43^. ABE7.10 was derived from TadA*6-dCas9 variants that harbor W23L/R, P48A, and R152H/P mutations after seven rounds of evolution and engineering. These mutations abrogate base-specific enzyme–DNA interactions to broaden target sequence compatibility, thereby enhancing ABE7.10-mediated base-editing efficiency at targets that contain multiple A residues. Subsequently, ABE7.10 editor was evolved into ABEmax for more efficient base editing by improving nuclear localization, maximizing protein expression, and ancestral reconstruction of the deaminase component^44^. To reduce off-target RNA-editing activity, miniABEmax was engineered by removing the wild-type TadA domain and altering amino acid residues (K20A/R21A or V82G) within the mutant TadA domain of ABEmax to reduce the RNA-recognition capability^167^. Compared with ABEmax, miniABEmax has lower Cas9-independent RNA-editing activity but maintains Cas9-assisted on-target DNA-editing function. Notably, the self-editing activity of miniABEmax was significantly lower than ABEmax. Self-editing by base editors can lead to the generation of truncated Cas9n with intact deaminase activity, thereby inducing off-target RNA editing but not on-target DNA editing.

The recent development of xCas9-derived base editors that recognize a broad range of PAM sequences has expanded the targeting scope in the genome^168,169^. Despite its broadened PAM compatibility, xCas9 retains high DNA specificity with minimal off-target activity. Subsequently, the base-editing efficiency of the xCas9-derived base editors (for example, xBE3 and xABE) was improved by fusing the BPNLS or Gam (or both) to the N terminus of xCas9-derived base editors (for example, BPNLS-Gam-xBE3 and BPNLS-xABE)^45^. Biparticle nucleus localization signal (BPNLS) enables efficient localization of base editors to the nucleus, while the Gam protein of bacteriophage Mu minimizes the production of undesired by-products upon base editing. More recently, NG-BE4max and NG-ABEmax base editors were generated by fusing a newly engineered SpCas9-NG variant to BE4max or ABEmax base editor^170^. SpCas9-NG variant recognizes minimal NG PAMs more efficiently than the xCas9 variant, thereby enabling efficient base editing with expanded targeting scope. The targeting scope and editing window of base editors have also been expanded using circularly permuted and PAM-modified Cas9n variants such as CP-CBEmax and CP-ABEmax^158^. In 2020, SpRY, a near-PAMless SpCas9 variant, was successfully engineered to target almost all PAMs (NRN>NYN PAMs), thereby eliminating PAM recognition constrains^171^. To overcome potential undesirable off-target effects due to the relaxation of the PAM requirement, a high-fidelity SpRY variant (SpRY-HF1) with improved DNA targeting specificity was subsequently engineered. Compared with dCas9, xCas9, and SpCas9-NG, SpRY-derived base editors enabled robust editing of the vast majority of disease-relevant genetic variants in the genome.

To enrich base-edited cells and increase the base-editing efficiency, a double-check base-editing approach is employed to exert a selective pressure against non-edited cells (Figure 5G)^172^. In this case, an inducible active Cas9 with the same sgRNA as the base editor is employed. The cells with non-edited target bases remain vulnerable to Cas9 cleavage. Cas9-mediated double-strand breaks lead to the death of non-edited cells in prokaryotic cells. However, the NHEJ is the major outcome instead of cell death in mammalian cells. To enrich base-edited cells in eukaryote cells, several alternative reporter systems have been developed^173,174^. For example, a transient reporter that converts blue fluorescent protein (BFP) to GFP upon a base substitution was employed^173^. The GFP-positive cells can be subsequently sorted for enrichment of base-edited cells. Compared with reporters of transfection that reflect only transfection efficiency, this BFP-to-GFP conversion reporter provides more accurate information about base-editing efficiency. In another reporter system, an inactivated eGFP reporter was placed downstream of wild-type mCherry and a T2A site^174^. The eGFP fluorescence is restored by correcting a point mutation in eGFP transgene via base editing. In this way, mCherry allows the identification of successfully transfected cells, while eGFP enables the quantification of single base-editing efficiency. The eGFP- and mCherry-positive cells then can be sorted. These two fluorescent-based reporter systems also allow high-throughput evaluation and identification of small molecules and factors that influence base-editing efficiency. To generalize the applications of the reporter to different cell types and systems, a functional reporter system (GO system) was subsequently developed for enrichment of base-edited cells^175^. GO works by initiating protein expression and affecting protein translation of different reporter proteins through correction of a mutated start codon immediately downstream of a kozak sequence using a base editor. This flexible GO system has helped to expand the base-editing reporter toolbox because it can be used to induce the translation of an array of different reporters, including fluorescent proteins, antibiotic resistance, and luciferase.

### Prime editing

In 2019, a prime-editing strategy was developed to overcome the limitations of homology-directed gene correction and base editing^46^. Prime editing has lower off-target editing than Cas9 nuclease and displays higher editing efficiency with fewer by-products than the CRISPR/Cas9-mediated homologous recombination approach. Compared with homology-dependent approaches that introduce edits within 10 bp from the Cas9 cleavage site, prime editor can install point mutations at distances of more than 30 bp from the Cas9n nicked site^176^. Therefore, prime editor offers greater targeting flexibility than the homology-dependent approaches. Most importantly, prime editing enables correction of all types of substitutions such as transitions and transversions as well as small insertions and deletions without requiring double-strand breaks or exogenous donor DNA repair templates^177,178^. Prime editing could also complement base editing in the case of unwanted bystander edits from the presence of multiple cytidine or adenine bases within the editing window of base editors^176^. Furthermore, prime editing outperformed the base editor for bases positioned outside the center of the base-editing window and could be an alternative tool when the desired genomic DNA site is not targetable by the base editor.

**Figure 6.**
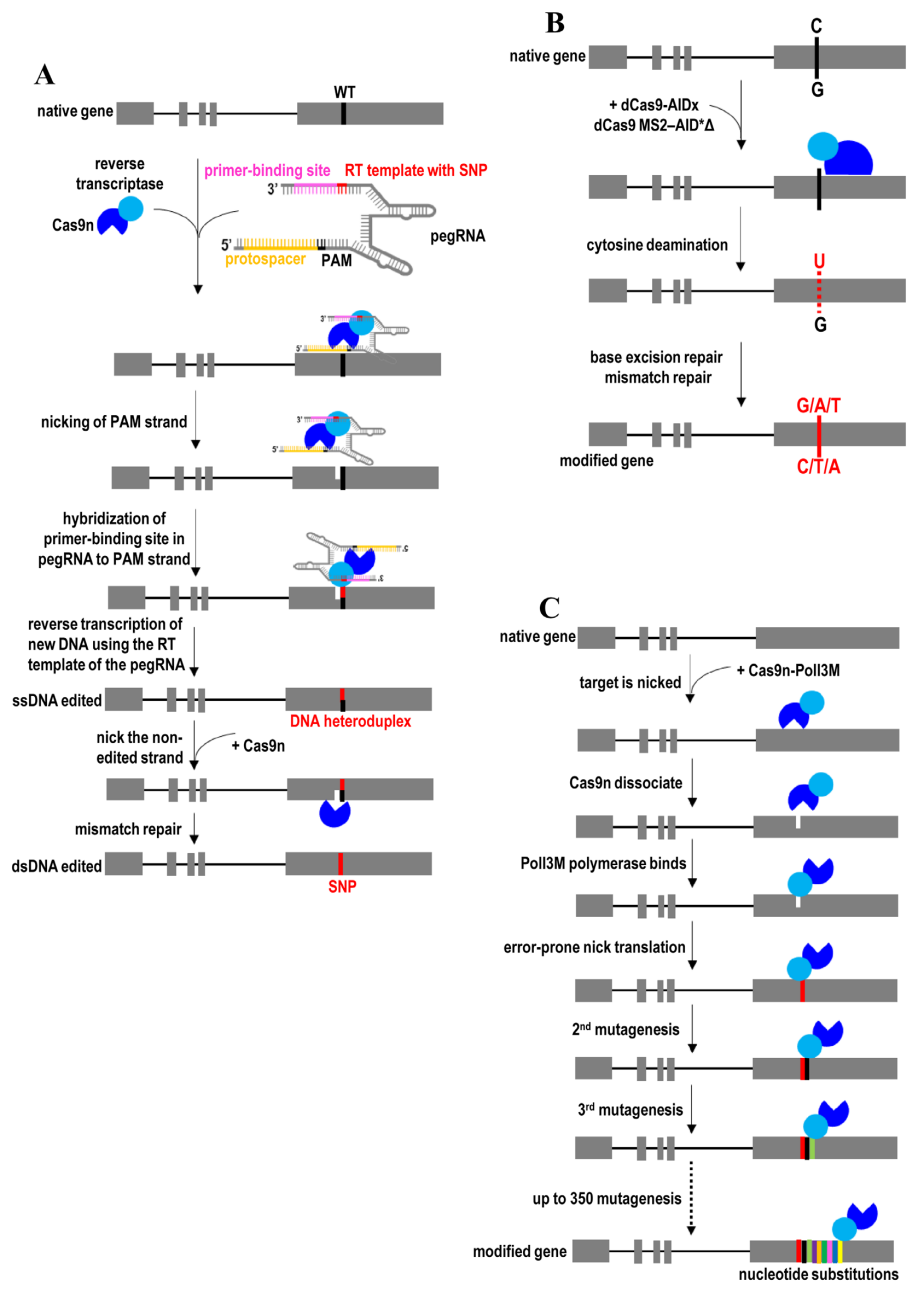
Prime editing and genetic diversification. (**A**) Gene correction by prime editing using Cas9n-RT complexed with the pegRNA. (**B**) Targeted mutagenesis by converting cytidines or guanines to the other three bases at desired loci using dCas9-AIDx. (**C**) Continuous diversifying all nucleotides within a tunable window length at a target locus using Cas9n-PolI3M. AID, activation-induced cytidine deaminase; Cas9n, Cas9 nickase; MS2, bacteriophage coat proteins; pegRNA, prime-editing guide RNA; PolI3M, fidelity-reduced variant of *Escherichia coli* DNA polymerase I (PolI) harboring the mutations D424A, I709N, and A759R; RT, reverse transcription; SNP, single-nucleotide polymorphism.

Prime editing uses a fusion protein consisting of Cas9n and an engineered RT. Importantly, a prime-editing guide RNA (pegRNA) that both specifies the target site of sgRNA and encodes the desired edit of the RT template is used to form complexes with Cas9n–RT. As a general principle, Cas9n–RT complexed with the pegRNA first binds and nicks the target DNA (Figure 6A). The resulting 3′ end of the nicked DNA strand hybridizes to the primer binding site in pegRNA. Then the RT template of the pegRNA is used to prime reverse transcription of new DNA bearing the desired edit. The desired genetic information is directly copied from an extension on the pegRNA to replace the target DNA in the genome. Equilibration between the edited 3′ flap and the unedited 5′ flap, cellular 5′ flap cleavage and ligation, and base excision repair will eventually lead to nucleotide substitutions at the target DNA site. Prime-editing efficiency is further increased by nicking the non-edited strand to induce DNA repair of that strand. This repair leads to the generation of duplex DNA containing the desired edit. The prime-editing strategy has been successfully applied in post-mitotic, terminally differentiated primary cells^46^. The efficiency of prime editing is determined largely by the design of the pegRNA such as the length of primer binding site and RT template as well as the GC content, primary sequence motifs, and secondary structures of the pegRNA^176^. Manipulation of DNA repair machinery to favor the replacement of the non-edited strand in the DNA heteroduplex could also improve the desired editing outcome.

Since prime editing and base editing do not employ any selection marker, their precise, scarless genome-editing events can be identified through the recently developed dinucleotide signature capture (DTECT) detection method^179^. The application of primer editor, similar to that of base editors, is limited to correcting point mutations or small genetic alterations. HDR- and NHEJ-based approaches are preferred to confer large DNA insertions or deletions. The addition of the desired edit of the RT template to the sgRNA may destabilize the extended sgRNA through the activity of intracellular RNA-degrading enzymes^180^. Therefore, it remains impractical to add a very long RT template to the sgRNA for large genetic alterations. Moreover, the RT template in pegRNA can be incorporated in the genome during reverse transcription. It is also a challenge to co-package Cas9n-RT and its pegRNA into a single AAV vector for prime editing *in vivo* because of large sizes of Cas9n-RT and the lengthened sgRNA. This AAV packaging issue can be addressed by using smaller Cas9n and RT enzymes.

### Genetic diversification

CRISPR technology has recently been harnessed for modifying multiple DNA bases simultaneously and enabling genetic diversification in living cells through targeted mutagenesis in the genome. Genetic diversification is useful for establishing complex libraries of genetic variants, enabling high-throughput screening of functional variants, evolving protein structure and function *in situ*, engineering antibodies, mapping protein–drug interactions, and cell-lineage tracking.

In 2016, two independent research groups developed the dCas9-AIDx fusion protein for converting cytidines or guanines to the other three bases at the desired loci (Figure 6B)^181,182^. When dCas9-AIDx is used together with multiple sgRNAs, it expands the editing window and generates more mutations in the protospacers. The dCas9-AIDx is created by fusing dCas9 to the activation-induced cytidine deaminase (AID). AID directs somatic hypermutation to a given genomic locus by deamination of cytosine to uracil. After deamination, point mutations are generated through misread of the uracil–guanine mismatch, base-excision repair of the uracil, or mismatch-repair pathway to generate transitions and transversions near the lesion^182^. Targeted mutagenesis efficiency is further improved by incorporating two MS2 hairpin-binding sites to the sgRNA for recruiting multiple copies of the hyperactive AID variant (AID*Δ) to each dCas9^182^. Besides, targeted mutagenesis specificity of dCas9-AIDx is improved by blocking uracil DNA glycosylase^181^. Coupled with uracil-DNA glycosylase inhibitor, dCas9-AIDx specifically converts cytidines to thymines in the protospacer. AIDx has also been fused to Cas9 nickase for optimal nucleotide conversion^181^. Therefore, these targeted mutagenesis strategies create a diverse repertoire of point mutations *in situ*, dismissing the need to use error-prone PCR to create mutations *in vitro* or design a large number of DNA oligos for incorporation into a genomic locus. It also introduces a large spectrum of point mutations without generating insertions and deletions observed with active Cas9. Nevertheless, Cas9n-AIDx can induce double-strand DNA breaks when designing multiple sgRNAs to target different target DNA sites in the genome^181^. To improve the efficiency of base editing in GC contexts and reduce bystander activity, AID-Cas9 was later evolved into the enhanced AID-Cas9 fusion (eAID-BE4max)^183^.

In 2018, another strategy that uses CRISPR-guided DNA polymerases (Cas9n-PolI3M) was developed for genetic diversification in living cells^47^. Compared with dCas9-AIDx-based targeted mutagenesis approaches (editing window within five nucleotides)^181,182^, Cas9n-PolI3M is capable of continuously diversifying all nucleotides within a tunable window length (up to 350 nucleotides) at user-defined loci^47^. Therefore, Cas9n-PolI3M generates a larger spectrum of substitution mutations than dCas9-AIDx for large-scale genetic screenings. Cas9n-PolI3M consists of a CRISPR-guided nickase (Cas9n) and a fused nick-translating DNA polymerase (PolI3M)^47^. The targeted mutation rate of Cas9n-PolI3M was enhanced by creating three mutations (K848A, K1003A, and R1060A) to Cas9n to promote the dissociation of Cas9n from DNA after nicking the target locus. In addition, a more processive DNA polymerase variant, PolI3M, was engineered to increase the length of the editing window and provide a higher targeted mutation rate than the wild-type DNA polymerase. As a general principle, Cas9n first nicks the target locus, resulting in a single-strand break in DNA (Figure 6C). Then Cas9n from the Cas9n-PolI3M complex dissociates, followed by the binding of PolI3M to the nicking site. Using this nicked DNA as an initiation site, PolI3M performs error-prone nick translation via strand displacement synthesis and cleavage of the displaced strand by the flap endonuclease domain. The resulting ligatable nick at the start site can undergo targeted mutagenesis. Finally, PolI3M continues to mutagenesis other nucleotides in the vicinity of the start site or nick site with an editing window of up to 350 nucleotides. Therefore, Cas9n specifies the start site of the editing window for PolI3M, while the mutagenesis window length and mutation rate are determined by PolI3M. This strategy enables multiplexing and continuously diversifying all nucleotides within user-defined genomic loci. Also, this strategy is more efficient than the homology-directed oligonucleotide integration approach. Simultaneously diversifying multiple loci through co-expression of multiple gRNAs is useful for studying epistatic interactions. In this case, multiple gRNAs targeting the same strand are used to nick the same strand to avoid double-strand breaks.

### RNA editing

Pathogenic point mutations can be directly corrected in RNA transcripts instead of mutagenesis of the genomic DNA^49,184,185^. Cas9 activity poses the risk of unanticipated chromosomal rearrangement at the targeted sites and permanent, off-target mutagenesis to DNA^120^. In this case, RNA base-editing technology offers a safer strategy by directly editing the pathogenic transcripts without the loss of genomic information. Because RNA editing is transient, it ensures that no by-products or unwanted mutations are generated in genomic DNA that can be passed on to daughter cells during mitosis. It also useful for curing diseases without a genetic origin and mimicking genetic variants that provide a health advantage^186^. Because dCas13 has no targeting sequence constraints and no motif preference surrounding the target base, RNA base-editing technology helps to circumvent the issue associated with the limited targeting sites of DNA base editors. RNA base-editing technology does not rely on endogenous DNA repair pathways; therefore, it mediates efficient RNA editing even in post-mitotic cells.

**Figure 7.**
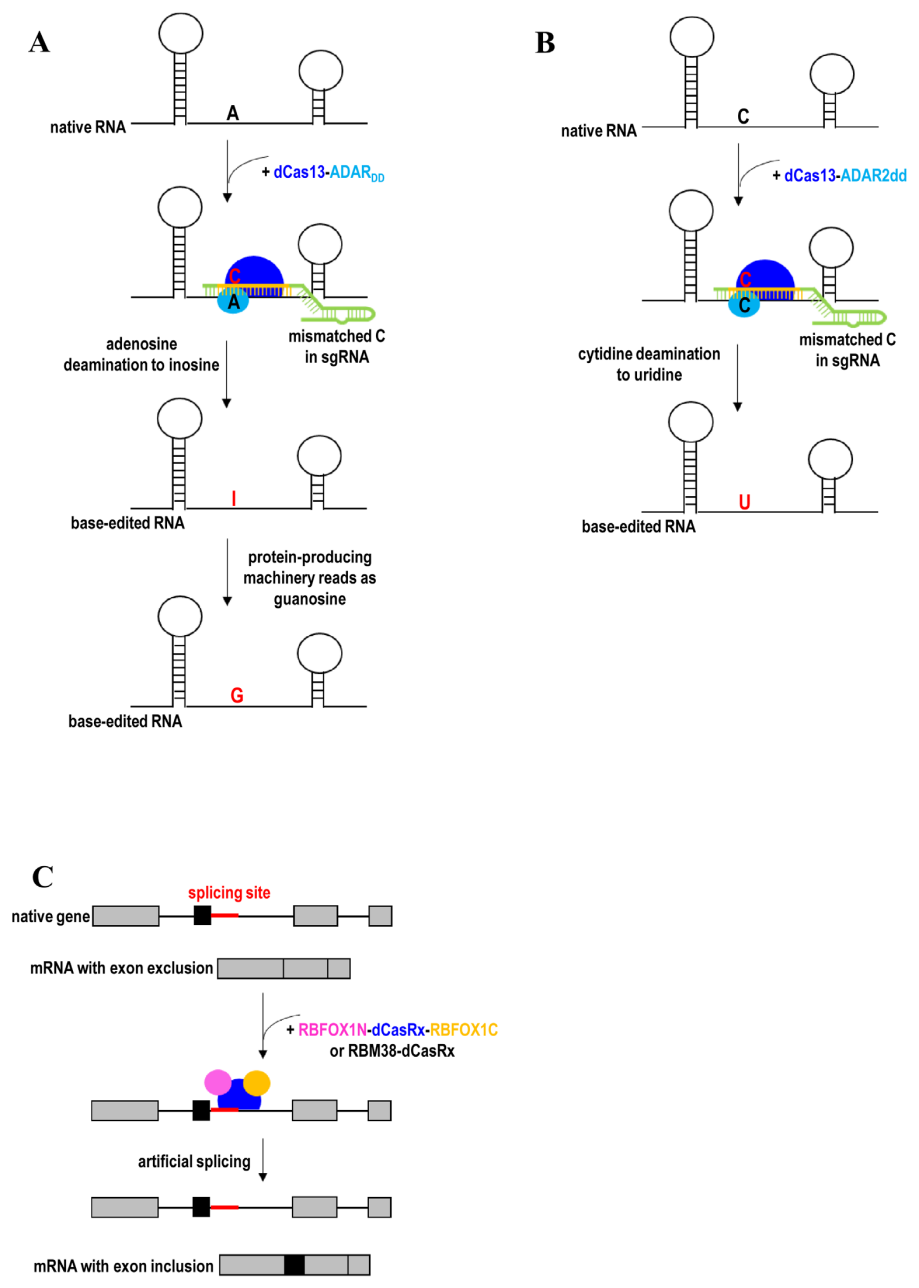
RNA editing. (**A**) A-to-G substitution by directing adenosine-to-inosine deaminase activity to endogenous RNA transcripts using dCas13-ADAR_DD_. (**B**) C-to-U substitution by directing cytidine-to-uridine deaminase activity to endogenous RNA transcripts using dCas13-ADAR2dd. (**C**) Exon inclusion to the mRNA by using a fusion of dCasRx and splicing regulatory domains. ADAR, adenosine deaminase; RBFOX1, RNA-binding protein fox-1 homolog; RBM38, RNA-binding motif protein 38.

dCas13-ADAR2 has been employed to direct adenosine-to-inosine deaminase activity to endogenous RNA transcripts (Figure 7A)^49^. dCas13 is capable of binding mRNA in a nucleic acid–programmed manner, while ADAR2 (adenosine deaminase acting on RNA type 2) deaminates adenosines mispaired with cytidine bases in RNA duplexes without any protein co-factors. The deaminase domain of ADAR2-containing hyperactivating mutations (ADAR_DD_) was later fused to dCas13b to enhance RNA-editing efficiency. The editing specificity of dCas13b-ADAR2_DD_ was improved by structure-guided protein engineering of ADAR2_DD_ (E488Q/T375G) to destabilize its binding to off-target RNA. Owing to the exogenous nature of dCas13b-ADAR2_DD _fusion proteins, host immune responses may compromise the efficacy of the RNA editing and cause side effects *in vivo*. The dCas13b-ADAR2_DD _fusion protein is also too large for packaging into the limited payload capacity of AAV vector. About two years later, dCas13b-ADAR2_DD_ was refined to improve RNA-editing specificity and overcome immunogenicity issues associated with ectopic overexpression of ADAR by recruiting endogenous ADAR using short engineered ADAR-recruiting RNAs (arRNAs)^48^. The arRNA is a long-guide RNA (≥71 nucleotides) that could anneal with the target transcript to form dsRNA substrate. The dsRNA substrate then recruits endogenous ADAR proteins for targeted editing. Placing the A-C mismatch in the middle region of arRNAs results in the highest RNA-editing efficiency, while the addition of multiple A-G mismatches to each arRNA minimizes off-target editing. To further enhance RNA-editing efficiency, arRNA is fused with an ADAR-recruiting scaffold to recruit multiple copies of ADAR protein to target RNAs. A similar arRNA scaffold can also be used to recruit endogenous ADAR proteins to edit the genetic materials of RNA virus such as coronavirus in order to restrict viral propagation in the host. In fact, there was evidence for host-dependent RNA editing in the transcriptome of coronaviruses from coronavirus-infected patients^187^.

To complement adenosine-to-inosine RNA-editing technology, a cytidine-to-uridine RNA editor was subsequently developed by directly evolving the adenine deaminase domain of ADAR2 (ADAR2dd) into a cytidine deaminase^50^. ADAR2dd then was fused to dCas13 for targeted RNA editing (Figure 7B). The specificity of dCas13-ADAR2dd was further improved via rational mutagenesis of ADAR2dd at residues (S375A) interacting with the RNA target. The RNA-editing specificity was also improved by introducing disfavorable guanine mismatches to the guide RNA. Apart from expanding the RNA-editing toolbox, the cytidine-to-uridine RNA editor is capable of multiplex RNA editing by having the capability of both adenosine-to-inosine and cytidine-to-uridine conversions.

More recently, CRISPR artificial splicing factors (CASFx) were developed for manipulation of gene splicing^188^. Although dCasRx alone was sufficient to promote exon exclusion by blocking access of splicing machinery^189^, dCasRx requires splicing regulatory domains to induce exon inclusion. Alternative slicing can be programmed using RBFOX1N-dCasRx-C or RBM38-dCasRx, a fusion of dCasRx and splicing factors (RBFOX1 or RBM38)^188^. The RNA-binding domain of splicing factors was substituted by dCasRx to derive the CASFx. For example, RBFOX1N-dCasRx-C or RBM38-dCasRx induces exon inclusion when bound downstream of the target exon (Figure 7C). In contrast, exon exclusion is induced when CASFx bind within a target exon. Therefore, this strategy can induce exon inclusion and exclusion to the mRNA without the need to modify the DNA sequence or introduce permanent changes in the genome. CASFx can also be applied transiently such that the alternative splicing is reversible.

Despite the reactiveness of RNA editing for research and therapeutic potential, RNA editing is much less efficient than gene editing^186^. Moreover, endogenous ADAR proteins are usually present in small amounts in most tissues for arRNA-based recruitment, thereby undermining RNA-editing efficiency in complex tissue^186^. The efficiency of RNA editing can be improved by using chemically modified sgRNA and a next-generation viral delivery vector or nanoparticle. For example, AAV delivery of exogenous RNA editor and guide sequence has enabled *in vivo* repair of mutant RNAs in mouse models of neurological disease^190^. Compared with gene editing, RNA editing also makes limited kinds of change to RNA. However, RNA-editing capabilities could be expanded through protein engineering to derive new RNA editors.

## Summary and conclusions

Various CRISPR-mediated homology-dependent and -independent gene knock-in and gene correction strategies have been developed. Multiplex, flexible, scarless gene insertion and replacement—together with the emerging tools for genetic diversification, prime editing, base editing, and RNA editing—can be achieved at high efficiency and specificity. We highlight structure-guided engineering of Cas9 variants that have greatly improved the gene-editing efficacy, specificity, editing window, and targeting scope. In addition, we comprehensively discuss factors involved in various DNA repair pathways enabling transgene integration and gene correction, such as Cas9 variants (for example, Cas9 nuclease, Cas9 nickase, Cpf1 nuclease, CasX, dCas9, or dCas13), fusion domains (for example, HDR enhancer, base editor, splicing effector, polymerase, or RT), donor type (for example, plasmid DNA, ssODN, PCR fragments, or homologous chromosome), length of homology arms (for example, none, 5–80 bp, or 500–1000 bp), number of sgRNAs (for example, single-cut or double-cut on donor and genomic DNA), DNA cleavage (for example, double-strand break, single-strand nick, or no cleavage), and cleavage pattern (for example, blunt or staggered cut).

We discuss the choice of gene correction approaches depending on the nature of the mutations (coding, non-coding, or splicing variants), types of mutation (substitution, deletion, or duplication), copy of alleles for modification (monoallelic or biallelic), and editing outcome (scarless modification or insertion of the selection marker to the genome for the enrichment of edited clones). We also discuss the choice of transgene knock-in approaches determined by the transgene size, donor design (the length of homology arms), plasmid donor conformation (circular, single-cut linearized, or double-cut linearized), chromatin accessibility of insertion site (open or closed chromatin), and the purpose of transgene knock-in (for overexpression, gene tagging, exon skipping, exon inclusion, gene knockout, gene replacement, or study of functional regulatory elements).

Finally, we discuss various strategies to address the potential immunogenicity associated with exogenous CRISPR fusion proteins. These include strategies to recruit endogenous catalytic domains (for example, base editor, HDR enhancer, RNA editor, or chromatin modifier) for gene knock-in and gene correction and controlled timing of CRISPR/Cas9 delivery and expression to minimize the off-target effects, toxicity, and immunogenicity associated with CRISPR components. Immunogenicity issue can also be circumvented by transient modulation of the genes involved in immunity by using a CRISPR-based synthetic repressor^191^. Other strategies to minimize undesired off-target mutagenesis are using light-activated, chemical-inducible, or multiple-input logic gate genetic circuits to spatiotemporal and conditional control of CRISPR/Cas9 activities^192^; cell type- or tissue-specific promoters and delivery vehicles (for example, specific viral serotypes^16,17,193^ or ligand-targeted cargos^192^), engineered high-fidelity Cas9 variants^194–200^, and rational chemical modifications to the sgRNA^192^; a chimeric DNA-RNA guide^201^; and engineered truncated sgRNA^202,203^ and sgRNA secondary structures^204^. Onsite mutagenesis and heterogeneity can also occur in the uncorrected population of cells following CRISPR/Cas9-induced double-stranded DNA breaks^99^. We also emphasize the importance of examining the potential unwanted head-to-tail insertions of donor DNA templates into the genome by HDR or NHEJ mechanisms (or both) before precisely edited alleles can be correctly identified^205^ and how chemical modification of both ends of the donor can help overcome this issue by enabling only one whole copy of the desired DNA to be inserted into the genome. In this fast-moving field of CRISPR-mediated gene editing, we hope to provide comprehensive information on recent developments to a broad scientific community for facilitating the application of this versatile technology.
